# Mechanical Performance Degradation of ECO EPDM Elastomers in Acidic Fuel Cell Environments

**DOI:** 10.3390/ma18092071

**Published:** 2025-04-30

**Authors:** Daniel Foltuț, Viorel-Aurel Șerban

**Affiliations:** 1 Department of Materials and Manufacturing Engineering, Politehnica University of Timișoara, Mihai Viteazul Blv., 300222 Timisoara, Romania; viorel.serban@upt.ro; 2 Technical Sciences Academy of Romania, B-dul Dacia 26, 030167 Bucuresti, Romania

**Keywords:** ECO EPDM, circular carbon black, recycled carbon black, fuel cell sealing, sulfuric acid aging, thermal degradation, swelling behavior, mechanical properties, SEM-EDS, sustainable elastomers

## Abstract

Sustainable ethylene propylene diene monomer (EPDM) elastomers are gaining traction as eco-friendly sealing materials in fuel cell applications. This study evaluates the mechanical degradation behavior of two ECO EPDM formulations—one reinforced with circular carbon black (CCB EPDM), and the other with recycled carbon black (RCB EPDM)—under conditions representative of acidic fuel cell environments. The samples underwent thermal aging at 90 °C for 1000 h, and were immersed in aqueous H_2_SO_4_ solutions of varying concentrations (1 M, 0.1 M, and 0.001 M) for 1000 h at the same temperature. Gravimetric and volumetric swelling measurements revealed that RCB EPDM experienced significantly higher mass and volume uptake, particularly at intermediate acid concentration, indicating greater susceptibility to fluid ingress. Mechanical testing, including measurement of tensile strength, Shore A hardness, and IRHD microhardness, showed that while RCB EPDM exhibited higher initial strength, it degraded more severely under thermal and acidic exposure. SEM-EDS analysis revealed microstructural damage and compositional changes, with RCB EPDM displaying more pronounced oxidation and surface erosion. In contrast, CCB EPDM demonstrated greater retention of mechanical integrity, greater dimensional stability, and lower variability across aging conditions. These findings highlight the advantages of circular carbon black in enhancing the durability of ECO EPDM compounds in acidic and thermally dynamic fuel cell environments.

## 1. Introduction

Proton exchange membrane fuel cells (PEMFCs) have emerged as a competitive energy conversion technology in automotive and stationary power systems, due to their high efficiency, compactness, and low environmental impact [1,2,3,4]. Within PEMFC stacks, elastomeric seals are critical components that prevent leakage of hydrogen, oxygen, and coolant, while maintaining electrical isolation and mechanical compression. These seals operate under complex, aggressive conditions—elevated temperatures (typically 60–90 °C), high relative humidity (50–100%), and prolonged exposure to mildly acidic aqueous environments due to the formation of sulfuric acid and fluoride-containing condensates. During operation, sulfuric acid may form as a result of membrane degradation, catalyst reactions, or accumulation of impurities in the water management system. Additionally, fluorinated compounds can break down, generating acidic species that condense within the cell environment. These acidic byproducts can migrate toward the elastomeric seals. This interaction poses risks of hydrolysis, oxidation, and filler leaching over time. Therefore, assessing the behavior of EPDM-based materials in sulfuric acid environments is directly relevant to evaluating their long-term durability for PEMFC applications [2,5,6].

Among commercially used elastomers, ethylene propylene diene monomer (EPDM) rubber remains a leading material for fuel cell sealing applications, due to its thermal stability, flexibility, and inherent resistance to ozone, oxidation, and polar media [2,7,8]. Compared to other sealing materials, such as silicone rubber, fluorinated elastomers, and thermoplastic vulcanizates (TPVs), EPDM has demonstrated lower compression set, better chemical resistance, and greater long-term sealing performance [2,9]. However, extended operation in PEMFC conditions can still lead to chemical and mechanical degradation processes. These include oxidative chain scission, de-crosslinking, surface cracking, and leaching of ionic species—processes that compromise mechanical integrity and dimensional stability [3,5,6].

Recent studies have focused on the development of more sustainable EPDM formulations by incorporating alternative fillers, such as circular carbon black (CCB) and recycled carbon black (RCB), derived from end-of-life tires through pyrolysis and other post-treatment processes [10,11]. While circular carbon black can offer particle morphology and performance characteristics comparable to those of furnace black, recycled carbon black often shows higher variability, and may affect crosslinking density and surface reactivity [7,11]. These material differences can have pronounced effects on aging behavior, especially under the acidic and thermal stress typical of PEMFC operation [3,12,13,14].

The degradation behavior of elastomeric compounds in simulated fuel cell environments has been investigated using various immersion aging protocols. Foltuț et al. [12] subjected EPDM, TPV, and ECO TPV specimens to acidic environments of 0.001 M, 0.1 M, and 1 M H_2_SO_4_ at 90 °C for 1000 h. EPDM showed the least dimensional change, with a volume increase of only +1.64% in 1 M H_2_SO_4_, compared to +4.87% in TPV and +6.50% in ECO TPV. Weight gain trends mirrored this behavior. Surface degradation phenomena, such as microcracking, were observed in ECO TPV and, to a lesser degree, in TPV, while EPDM largely preserved its surface morphology [2,12].

Mechanical testing further supported EPDM’s relative stability: tensile strength retention after acidic aging remained high (from 18.84 MPa in fresh state to 16.7–18.1 MPa), and elongation at break showed only minor reductions (187% to 163–183%) across all acid concentrations [15]. In contrast, ECO TPV exhibited pronounced mechanical degradation, particularly in high-acid conditions, with up to 15% tensile strength loss and increased surface embrittlement. Complementary DSC analysis revealed minor shifts in thermal transitions in aged EPDM, while ECO TPV displayed marked peak broadening and displacement, indicating structural destabilization [16].

Aside from mechanical and dimensional changes, ionic leaching and chemical stability are key considerations for seal materials in PEMFCs. A hot water extraction test performed by Foltuț et al. [12] showed that EPDM remained below the 5 μS/cm conductivity threshold for over 100 h of immersion, while ECO TPV rapidly exceeded this value, indicating more aggressive ion release. SEM and EDS analysis further confirmed the migration of polar fillers and additives in TPV and ECO TPV, whereas EPDM retained a more chemically inert surface [2,3].

In view of these findings, a more detailed evaluation of next-generation EPDM systems filled with circular or recycled carbon black is necessary to understand their long-term mechanical and chemical stability under conditions simulating the PEMFC environment. While baseline EPDM has proven to be robust, the implications of replacing traditional filler systems with sustainable alternatives must be thoroughly characterized.

The present study investigates the mechanical degradation behavior of two EPDM elastomers—one containing circular carbon black (CCB EPDM) and one containing recycled carbon black (RCB EPDM)—after prolonged exposure to acidic aqueous environments. Through a combination of mass and volume change measurements, mechanical property evaluation (tensile, Shore A hardness, microhardness), and surface analysis (SEM), the goal is to determine the material-specific trends in performance retention and failure mechanisms. This work builds upon previous investigations by incorporating sustainable filler systems while maintaining a testing framework aligned with real PEMFC operational stressors.

## 2. Materials and Methods

### 2.1. Materials

The materials investigated in this study were two EPDM compounds, referred to hereafter as CCB EPDM and RCB EPDM (produced by Arlanxeo, Geleen, The Netherlands) respectively. Both materials are based on a peroxide-cured EPDM matrix, but differ in the type of carbon black filler used. CCB EPDM incorporates circular carbon black, obtained through advanced pyrolysis and refinement of end-of-life tires, whereas RCB EPDM contains recycled carbon black, which is also recovered from waste tires, but typically involves less controlled processing, resulting in broader particle size distributions and increased variability.

The two materials were supplied as vulcanized sheets with identical base EPDM polymers and crosslinking systems, to isolate the influence of filler type on aging and mechanical performance. Basic mechanical and structural characteristics of the fresh materials are summarized in Table 1.

### 2.2. Aging Protocols

The aging process was designed to replicate the ambient chemical and thermal conditions encountered in fuel cell systems, with the objective of evaluating the robustness and degradation behavior of the EPDM materials under long-term exposure. Two separate aging procedures were employed: chemical aging in acidic aqueous solutions and thermal aging in dry heat.

For chemical aging, specimens of both EPDM compounds were fully immersed in aqueous sulfuric acid (H_2_SO_4_) solutions of three different concentrations: 1 M, 0.1 M, and 0.001 M, representing strongly acidic, moderately acidic, and mildly acidic environments, respectively. The pH values of the prepared solutions were approximately 0.1 for 1 M, 0.84 for 0.1 M, and 2.24 for 0.001 M H_2_SO_4_. Each specimen was submerged in solution within sealed borosilicate glass vessels and aged for 1000 h at 90 °C in a temperature-controlled oven. This protocol was intended to simulate proton exchange membrane fuel cell (PEMFC) operating environments, where acidic condensate can form and persist over time, leading to potential chemical degradation of sealing components.

In parallel, a separate set of samples was subjected to thermal aging under dry heat conditions. The materials were placed in a forced-air convection oven and exposed to 90 °C for 1000 h, without immersion in liquid media. This procedure was conducted to isolate and evaluate the effects of thermal exposure alone on the mechanical and microstructural stability of the materials, simulating fuel cell standby or elevated-temperature operating phases without active condensation.

Both chemical and thermal aging conditions were selected to accelerate degradation processes for comparison with earlier studies, and to provide insight into the environmental resilience of EPDM compounds containing either circular or recycled carbon black. Following aging, the materials were subjected to mechanical, thermal, and surface characterization to quantify changes in performance and structure.

### 2.3. Weight and Volume Measurements

The effects of chemical aging on the dimensional and mass stability of the EPDM materials were evaluated through gravimetric and hydrostatic measurements performed before and after immersion. Prior to aging, each sample was weighed in air, and then submerged in water, allowing for the calculation of its baseline volume through Archimedes’ principle. After aging, the same measurement procedure was repeated under identical conditions to evaluate changes due to fluid absorption, swelling, or degradation.

The rubber samples were rinsed with distilled water to remove residual solution, blotted with a lint-free cloth to remove bulk liquid, and air-dried in a desiccator with silica gel for several hours until a constant weight was achieved.

To determine the percentage mass change, the initial mass of the sample in air, $m_{0}$, and the final mass after immersion, $m_{i}$, were used. The mass variation was calculated according to the following equation:

(1) $$\Delta m_{100}=\frac{m_{i}-m_{0}}{m_{0}}\times 100$$

This expression quantifies the relative increase or decrease in specimen weight, indicating absorption or leaching behavior during aging.

The percentage change in volume was derived using the hydrostatic method, based on the specimen’s weight difference in air and in water. The volume of the sample was inferred from the buoyant force acting on it, using the following formula:

(2) $$\Delta V_{100}=\left(\frac{m_{i}-m_{i,w}}{m_{0}-m_{0,w}}-1\right)\times 100$$

where $m_{0,w}$ and $m_{i,w}$ are the apparent weights of the specimen in water before and after aging, respectively. Because no sinker was used, the expressions account solely for the specimen’s own submerged mass. This approach allowed for accurate tracking of swelling or shrinkage behavior because of long-term chemical exposure, without mechanical interference.

### 2.4. Mechanical Testing

The mechanical performance of the EPDM materials before and after aging was evaluated through tensile testing, Shore A hardness, and microhardness measurements. Tensile properties were assessed in accordance with ISO 37 [17], using Type 2 dumbbell-shaped specimens die-cut from the original vulcanized sheets. Each specimen had a gauge width of 4 mm and a thickness of 2 mm. Testing was carried out on an Instron universal testing machine, equipped with a video extensometer to accurately track elongation during loading. A crosshead speed of 500 mm/min was applied, and tests were conducted under standard laboratory conditions (23 °C, 50% RH). For each condition, a minimum of three replicates was tested. Stress–strain curves were recorded, and the average tensile strength and elongation at break were calculated for comparison across all aging conditions.

### 2.5. Scanning Electron Microscopy and Energy-Dispersive X-Ray Spectroscopy (SEM-EDS)

The surface morphology and elemental composition of the EPDM materials were investigated using scanning electron microscopy (SEM) combined with energy-dispersive X-ray spectroscopy (EDS) to assess microstructural and chemical changes resulting from thermal and chemical aging. Samples were analyzed in their fresh, heat-aged, and 1 M sulfuric acid-aged states, corresponding to the most representative conditions in the fuel cell-relevant environment.

SEM imaging was performed using a Thermo Fisher Axia ChemiSEM system (Thermo Fisher Scientific, Waltham, MA, USA). Specimens were mounted on aluminum stubs using conductive carbon adhesive and sputter-coated with gold to ensure conductivity. Images were acquired using a CBS detector at an accelerating voltage of 10.00 kV, a working distance of 10.2 mm, and magnifications of 500× and 1000×. The horizontal field width (HFW) for 1000× images was approximately 414 µm, providing detailed visualization of surface topography, including filler dispersion, roughness, and aging-induced degradation features.

Elemental analysis was conducted using integrated EDS mapping and point analysis functions. Spectra were collected at 10.00 kV for a total acquisition time of 379 s, achieving a total count of 432,475, with an average count rate of 1140 cps. Elemental maps were acquired at a resolution of 768 × 512 pixels. Quantitative EDS data were reported as atomic % and weight %, with key elements including carbon (C), oxygen (O), sulfur (S), and magnesium (Mg). In the acid-aged samples, increases in oxygen and sulfur content were tracked to assess oxidation and sulfur uptake due to chemical degradation. Trace elements such as magnesium were also monitored to detect filler leaching or migration.

## 3. Results

### 3.1. Weight and Volume Changes

**Figure 1:**
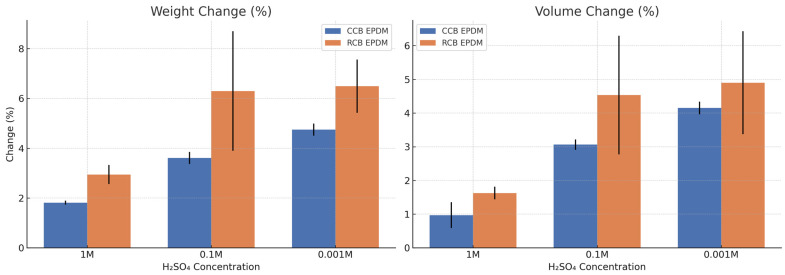
Weight and volume changes (%) of CCB EPDM and RCB EPDM after 24 h immersion in aqueous H_2_SO_4_ solutions of varying concentrations (1 M, 0.1 M, 0.001 M) at 80 °C. Error bars represent standard deviation across three replicates.

Both ECO EPDM formulations exhibited measurable swelling, with trends clearly influenced by acid concentration and formulation type. As expected, weight and volume increases were more pronounced at lower H_2_SO_4_ concentrations (0.1 M and 0.001 M) for both materials, as highlighted in Figure 1. However, RCB EPDM consistently showed higher swelling responses compared to CCB EPDM across all conditions. At 1 M H_2_SO_4_, CCB EPDM exhibited an average weight increase of 1.82% and volume increase of 0.97%, while RCB EPDM showed increases of 2.95% and 1.63%, respectively. The difference widened at 0.1 M, at which concentration CCB EPDM’s changes reached 3.61% for weight and 3.06% for volume, while RCB EPDM’s changes rose to 6.30% and 4.53%, respectively. At the lowest concentration (0.001 M), RCB EPDM again swelled more, with a 6.50% weight increase and a 4.90% volume increase, compared to 4.75% and 4.15% for CCB EPDM.

These results indicate that RCB EPDM is more susceptible to fluid uptake, likely due to a more open or polar filler structure associated with the recycled carbon black. The pronounced swelling of RCB EPDM in 0.1 M H_2_SO_4_ suggests that under moderately acidic conditions, the material’s structure allows for greater water and ion penetration, possibly due to higher filler dispersion variability or pore development.

Conversely, CCB EPDM demonstrates more stable behavior, with relatively linear increases in swelling, implying improved network integrity or lower permeability under acidic conditions. The observed standard deviations further support that RCB EPDM displays greater variability, especially at 0.1 M, potentially reflecting microstructural inconsistency in the recycled filler system.

### 3.2. Tensile Testing

**Figure 2:**
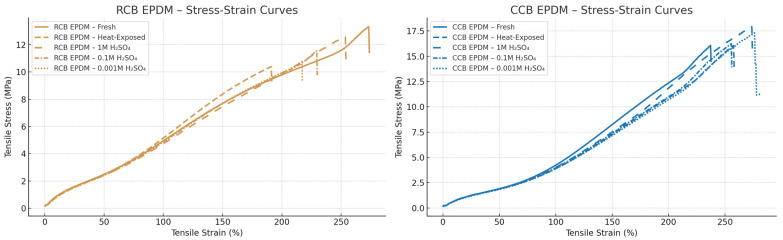
Stress–strain curves for RCB EPDM (**left**) and CCB EPDM (**right**) under fresh, heat-aged, and acid-aged conditions.

The tensile behavior of the two ECO EPDM materials—CCB EPDM and RCB EPDM—was evaluated under fresh, heat-exposed, and acid-aged conditions. Representative stress–strain curves were averaged from multiple replicates per condition, and strain was plotted as the percentage elongation (Figure 2). The mechanical performance was strongly influenced by both the formulation and the exposure environment.

Under fresh conditions, RCB EPDM exhibited approximately 12% higher average tensile strength than CCB EPDM, but also around 25% lower elongation at break, indicating a stiffer but less ductile behavior. This suggests that the recycled carbon black results in a more rigid but brittle network structure. Following thermal aging, both materials showed reductions in tensile performance, but RCB EPDM was more severely affected. Elongation at break for RCB EPDM decreased by more than 40% compared to the fresh state, whereas CCB EPDM retained over 70% of its original elongation, suggesting a more stable network structure and better thermal resilience in the CCB formulation.

**Figure 3:**
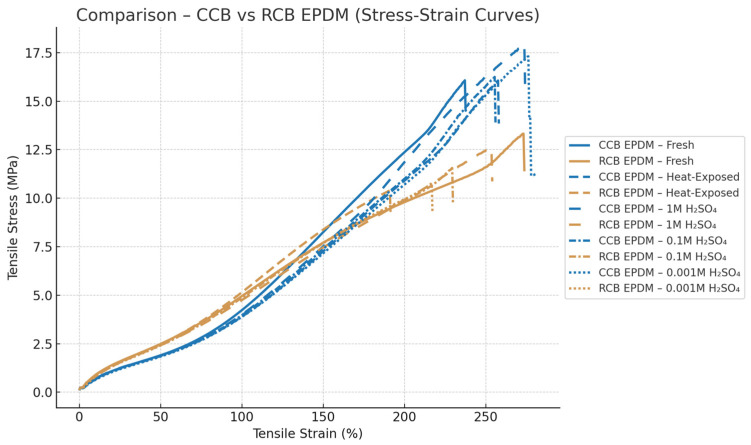
Comparative stress–strain behavior of CCB EPDM and RCB EPDM in fresh, heat-aged, and acid-aged states.

Chemical aging in sulfuric acid had a concentration-dependent impact on mechanical properties. In 1 M H_2_SO_4_, CCB EPDM maintained approximately 83% of its original tensile strength, while the tensile strength of RCB EPDM dropped to about 68%, along with a 30–35% reduction in its elongation. At 0.1 M, the contrast became even more pronounced: RCB EPDM lost nearly 45% of its strength and became notably brittle, while CCB EPDM showed a more moderate strength reduction of about 20%, maintaining smoother stress–strain transitions. At 0.001 M H_2_SO_4_, although both materials exhibited swelling, mechanical degradation was limited. CCB EPDM preserved over 90% of its original strength and ductility, whereas RCB EPDM still showed softening and greater variability. The comparison between the two materials can be seen in Figure 3.

Standard deviations in the tensile data further reflected the material differences. CCB EPDM exhibited lower variation across replicates, with typical standard deviations in the elastic region around 0.15–0.3 MPa, and less than 8% in elongation at break. In contrast, RCB EPDM displayed higher variability under chemical aging, with standard deviations exceeding 0.5 MPa and a larger spread in failure strain. The RCB EPDM stress–strain curves also ended more abruptly, suggesting premature failure, while the CCB EPDM curves retained smoother profiles under all conditions.

### 3.3. Hardness and IRHD Microhardness Changes

Hardness testing revealed clear differences in the aging response between the two EPDM compounds. Both Shore A and IRHD microhardness values changed after exposure to heat and acid, with RCB EPDM consistently showing higher absolute hardness values, but also a slightly greater softening trend across aging conditions. CCB EPDM exhibited lower initial hardness, but demonstrated more consistent performance under chemical and thermal stress.

**Figure 4:**
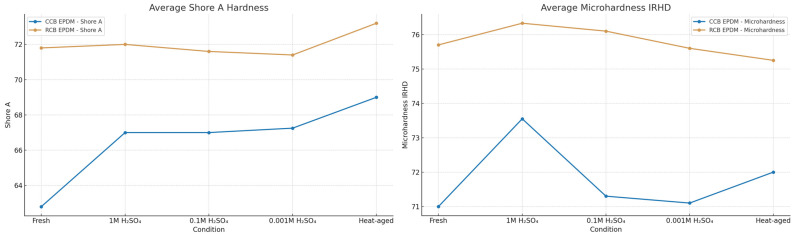
Average Shore A hardness (**left**) and IRHD microhardness (**right**) of CCB EPDM and RCB EPDM under fresh, heat-aged, and acid-aged conditions (1 M, 0.1 M, and 0.001 M H_2_SO_4_, 90 °C, 1000 h).

Figure 4 highlights the Shore A hardness and IRHD microhardness changes after the conditioning in the different media.

In the fresh state, RCB EPDM showed an average Shore A hardness of approximately 72, while that of CCB EPDM was measured to be around 63. A similar trend was observed in IRHD microhardness, with RCB EPDM’s reaching 76 and CCB EPDM’s reaching around 71. After 1 M H_2_SO_4_ exposure, both materials softened slightly.

CCB EPDM’s Shore A increased slightly to 67 and its IRHD increased to 73.5, while RCB EPDM showed a modest increase in IRHD (76.5) and stable Shore A (~72). Notably, CCB EPDM maintained stable Shore A values across all acid concentrations, while RCB EPDM showed a minor dip at 0.1 M H_2_SO_4_. After heat aging, both materials exhibited a recovery in surface hardness, with CCB EPDM’s values reaching nearly 69 for Shore A and 72 for IRHD, and RCB EPDM’s values peaking at 73 Shore A and 75.5 IRHD.

### 3.4. SEM-EDS Results

The surface morphology and elemental composition of both CCB EPDM and RCB EPDM were evaluated using scanning electron microscopy (SEM) and energy-dispersive X-ray spectroscopy (EDS) across fresh, heat-aged, and acid-exposed conditions.

**Figure 5:**
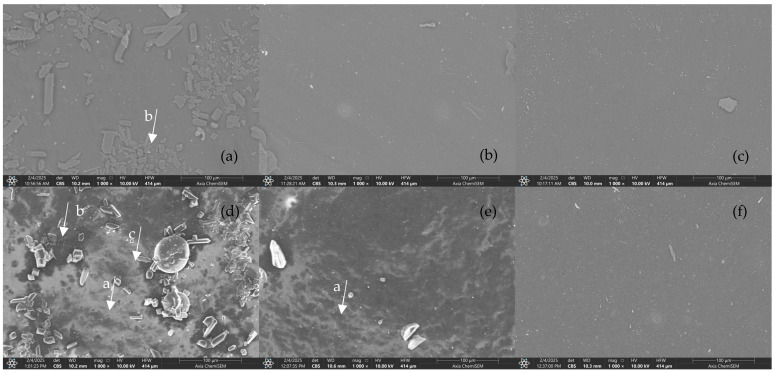
SEM micrographs of CCB EPDM (top row: **a**–**c**) and RCB EPDM (bottom row: **d**–**f**) at 1000× magnification under different conditions: (**a**,**d**) fresh samples, (**b**,**e**) heat-exposed (HE) samples, and (**c**,**f**) samples aged in 1 M H_2_SO_4_ solution.

**Figure 6:**
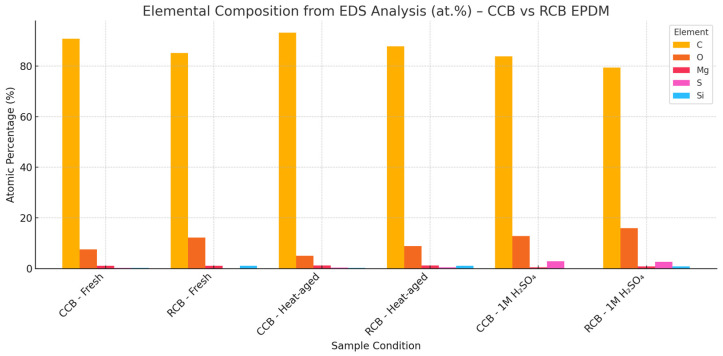
Elemental composition of RCB and CCB EPDM samples from EDS analysis.

In the fresh state, SEM images (Figure 5) of both materials showed smooth, uniform surfaces with well-dispersed filler phases. EDS analysis (Figure 6) confirmed the expected high carbon content in both samples—90.8 at.% for CCB EPDM and 85.1 at.% for RCB EPDM—alongside low oxygen levels and trace amounts of magnesium, silicon, sulfur (in CCB EPDM), and zinc (in RCB EPDM). The presence of Zn and Si in RCB EPDM is associated with the recycled filler origin, while the slightly higher oxygen in RCB EPDM may reflect surface oxidation or residual contamination from previous use.

Figure 5 provides detailed SEM micrographs comparing the surface morphologies of fresh and aged EPDM samples. In Figure 5d, representing the fresh RCB EPDM, clear evidence of surface porosity (marked as ‘a’) can be observed. These porous regions manifest as darker areas featuring distinct cavities and voids. Such porosity could potentially impact the material’s performance in harsh environments, influencing aspects like fluid absorption and mechanical durability.

In the same image (Figure 5d), an open filler structure is evident (marked as ‘b’). This structure is characterized by loosely aggregated filler particles, separated by noticeable gaps. A similar open filler structure is also present in fresh CCB EPDM (Figure 5a), demonstrating clear particle dispersion. Additionally, areas indicative of matrix erosion (marked as ‘c’), with irregular textures and signs of localized polymer degradation, are visible in fresh RCB EPDM. Such open structures and matrix erosion sites could lead to distinct physical properties, potentially influencing elastomer performance through differences in mechanical resilience, chemical resistance, or stress distribution within the polymer matrix.

Following heat exposure, both materials show minor surface roughening in their SEM images (Figure 5b,e), but no significant cracking or filler pull-out. In terms of composition, CCB EPDM exhibited a slight increase in carbon and a decrease in oxygen (93.2 and 5.0 at.%, respectively), suggesting limited thermal degradation. RCB EPDM showed a similar trend, with carbon rising to 87.7 at.% and oxygen decreasing to 8.9 at.%. The RCB sample also revealed the presence of sulfur (0.5 at.%) and calcium (0.1 at.%) post-aging, possibly from additive migration. Zinc and silicon remained at trace levels.

Exposure to 1 M H_2_SO_4_ caused visible degradation in both materials. The SEM micrographs (Figure 5c,f) reveal rough, irregular surfaces with microvoids and matrix erosion. For CCB EPDM, carbon dropped sharply to 83.8 at.%, while oxygen and sulfur rose to 12.8 and 2.9 at.%, respectively. RCB EPDM also showed carbon loss (79.4 at.%), with oxygen increasing to 16.0 at.% and sulfur to 2.6 at.%. Notably, the RCB EPDM surface retained detectable Zn and Si, but the magnesium content was slightly reduced in both formulations. These results suggest that both materials underwent oxidative and acid-induced degradation, but RCB EPDM showed greater carbon loss and oxygen uptake, indicating more severe chemical breakdown.

It should be noted that EDS analysis yields semi-quantitative elemental data, and the results are used here to indicate general trends in elemental migration and surface composition, rather than absolute quantification.

## 4. Discussion

The results of this study demonstrate distinct differences in the chemical and mechanical stability of the two ECO EPDM formulations when subjected to thermal and acidic aging conditions representative of fuel cell environments. The type of carbon black used—circular (CCB) versus recycled (RCB)—had a notable influence on swelling behavior, mechanical retention, surface hardness, and microstructural response.

Sulfuric acid is known to promote oxidative degradation of unsaturated elastomers through chain scission and incorporation of sulfoxide or carbonyl groups [6,8].

The swelling data clearly indicate that RCB EPDM is more susceptible to fluid uptake than CCB EPDM. Across all acid concentrations, RCB EPDM showed higher weight and volume increases, with the largest differences appearing at intermediate concentration (0.1 M H_2_SO_4_). This behavior may be linked to the microstructural and surface chemistry variability of the recycled carbon black, which can increase permeability and facilitate fluid diffusion [10,11]. In contrast, the more uniform particle size distribution and surface activity of circular carbon black likely contribute to a more stable filler–rubber interaction and reduced uptake. The consistent increase in swelling for both materials at lower acid concentrations also suggests a dilution-dependent osmotic uptake effect, where proton-rich but less concentrated solutions drive deeper water absorption over time [12].

Stress–strain analysis revealed that CCB EPDM consistently retained higher tensile strength and elongation at break across all aging conditions. While both materials experienced a decline in mechanical properties following exposure, RCB EPDM was more affected—particularly under 0.1 M H_2_SO_4_ conditions, which also coincided with its highest swelling values. This supports the interpretation that intermediate chemical stress can penetrate deeper into the matrix, disrupting the crosslinked structure more effectively when filler dispersion or compatibility is suboptimal. These findings align with earlier studies showing that EPDM exposed to sulfuric acid suffers from both chain scission and oxidative degradation, especially when the filler network is less integrated [6,8]. CCB EPDM’s smoother degradation trend and retention of tensile properties suggest greater filler–matrix compatibility, which may contribute to enhanced durability in fuel cell sealing applications.

Hardness measurements further support the trend of more stable aging behavior in CCB EPDM. Although RCB EPDM exhibited higher Shore A and microhardness values in the fresh state, its values showed slightly greater variability across aging conditions, particularly under acidic exposure. CCB EPDM, in contrast, displayed a more consistent hardness profile, with only minor changes after 1000 h in both sulfuric acid and thermal conditions. This surface stability complements its lower swelling and superior tensile retention, indicating reduced network disruption and less surface plasticization under aggressive environments.

SEM images and EDS analysis confirmed distinct differences in surface morphology and chemical stability. In RCB EPDM, the acid-aged surfaces showed more prominent microstructural roughness and particle pull-out, suggesting filler debonding or matrix erosion. EDS spectra showed an increase in sulfur content after immersion, along with elevated oxygen levels, indicative of surface oxidation and acid penetration. CCB EPDM surfaces, in contrast, remained smoother and more homogeneous, with less pronounced elemental changes. These findings suggest that the recycled filler system in RCB EPDM may facilitate pathways for acid ingress, contributing to the higher swelling and mechanical degradation observed.

The presence of Mg and other elements, such as Zn and Si, in RCB EPDM may have resulted from recycled filler composition, consistent with previous findings on carbon black from end-of-life tires [10]. In both materials, however, elemental sulfur was detected after aging, confirming interaction between the polymer matrix and the acid solution. The milder chemical changes in CCB EPDM again point to a more robust and chemically inert microstructure.

## 5. Conclusions

This study investigated the long-term thermal and chemical aging behavior of two sustainable EPDM materials—one filled with circular carbon black (CCB EPDM) and the other with recycled carbon black (RCB EPDM)—under conditions simulating acidic fuel cell environments. The results clearly show that the type of carbon black filler significantly influences swelling behavior, mechanical retention, hardness, and surface stability. RCB EPDM consistently exhibited higher weight and volume increases, greater variability in tensile strength, and more pronounced surface degradation compared to CCB EPDM. These trends suggest that recycled carbon black may introduce microstructural heterogeneity and increased permeability, making the material more vulnerable to acid-induced aging.

In contrast, CCB EPDM demonstrated more stable mechanical and dimensional behavior across all conditions, along with improved surface preservation and lower elemental migration under SEM-EDS analysis. These findings highlight the importance of filler morphology and compatibility in determining the durability of EPDM compounds in aggressive environments. The improved performance of CCB EPDM supports its potential use in long-life sealing applications for fuel cell systems, where resistance to both chemical and thermal stress is critical.

## Figures and Tables

**Table 1:** Properties of the EPDM compounds used in the study.

Property	CCB EPDM	RCB EPDM
Tensile strength (MPa)	15	12
Elongation at break (%)	271	262
Shore A hardness	77	75
Microhardness (IRHD)	47	45
Crosslinking system	Peroxide	Peroxide
Filler type	Circular carbon black	Recycled carbon black

## Data Availability

The original contributions presented in this study are included in the article. Further inquiries can be directed to the corresponding author.
